# Remote Outcomes with Poly-ε-Caprolactone Aortic Grafts in Rats

**DOI:** 10.3390/polym15214304

**Published:** 2023-11-02

**Authors:** Anna A. Dokuchaeva, Aleksandra B. Mochalova, Tatyana P. Timchenko, Elena V. Kuznetsova, Kseniya S. Podolskaya, Oxana A. Pashkovskaya, Natalya A. Filatova, Andrey A. Vaver, Irina Yu. Zhuravleva

**Affiliations:** Institute of Experimental Biology and Medicine, E. Meshalkin National Medical Research Center of the RF Ministry of Health, 15 Rechkunovskaya St., Novosibirsk 630055, Russia; mochalova_a@meshalkin.ru (A.B.M.); t_timchenko@meshalkin.ru (T.P.T.); ev_kuznetsova@meshalkin.ru (E.V.K.); podolskaya_k@meshalkin.ru (K.S.P.); o_pashkovskaja@meshalkin.ru (O.A.P.); filatova_n@meshalikn.ru (N.A.F.); vaver_a@meshalkin.ru (A.A.V.); zhuravleva_i@meshalkin.ru (I.Y.Z.)

**Keywords:** PCL, biodegradation, in vivo rat model, vascular scaffolds, electrospinning, abdominal aorta replacement

## Abstract

Poly-ε-caprolactone ((1,7)-polyoxepan-2-one; PCL) is a biodegradable polymer widely used in various fields of bioengineering, but its behavior in long-term studies appears to depend on many conditions, such as application specificity, chemical structure, in vivo test systems, and even environmental conditions in which the construction is exploited in. In this study, we offer an observation of the remote outcomes of PCL tubular grafts for abdominal aorta replacement in an in vivo experiment on a rat model. Adult Wistar rats were implanted with PCL vascular matrices and observed for 180 days. The results of ultrasound diagnostics and X-ray tomography (CBCT) show that the grafts maintained patency for the entire follow-up period without thrombosis, leakage, or interruptions, but different types of tissue reactions were found at this time point. By the day of examination, all the implants revealed a confluent endothelial monolayer covering layers of hyperplastic neointima formed on the luminal surface of the grafts. Foreign body reactions were found in several explants including those without signs of stenosis. Most of the scaffolds showed a pronounced infiltration with fibroblastic cells. All the samples revealed subintimal calcium phosphate deposits. A correlation between chondroid metaplasia in profound cells of neointima and the process of mineralization was supported by immunohistochemical (IHC) staining for S100 proteins and EDS mapping. Microscopy showed that the scaffolds with an intensive inflammatory response or formed fibrotic capsules retain their fibrillar structure even on day 180 after implantation, but matrices infiltrated with viable cells partially save the original fibrillary network. This research highlights the advantages of PCL vascular scaffolds, such as graft permeability, revitalization, and good surgical outcomes. The disadvantages are low biodegradation rates and exceptionally high risks of mineralization and intimal hyperplasia.

## 1. Introduction

Biodegradable polymers are widely applied in tissue engineering [1,2,3,4,5]. These materials provide easy processing, modification and device fabrication, diversity of chemical and physical properties, and biocompatibility. However, their variability is accompanied by a high specificity of obtained polymer compounds. Even when considering compositions from a single monomer, the difference in their structural, chemical, and biological features can be vast [6,7,8,9]. Due to the fact that minor changes in the chemical structure of polymers can affect their physical and biological properties, it is difficult to predict their in vivo performance, much less to collect reliable data about them.

PCL ((1,7)-polyoxepan-2-one, poly-ε-caprolactone) is a polymer of the polyester family, synthesized from ε-caprolactone by ring-opening polymerization with a biologically safe product of degradation, 6- hydroxyhexanoic acid, and well-known ways of its utilization in the organism. PCL can be degraded either by hydrolytic or enzymatic pathways [7,8,9]. This polymer is used for different purposes in tissue engineering, including vascular grafting [10,11,12,13,14]. Although a lot of research has already been performed on this polymer, different research groups give diverse data concerning PCL in vivo performance as vascular grafts [15,16].

One of the most common ways of polymeric vascular scaffold fabrication is electrospinning. This technology, based on the influence of a high voltage electric field on a stream of a conductive solution or melt (Figure S1), is the easiest way to produce non-woven nanofiber scaffolds with an adjustable fiber diameter, network pattern, porosity, and shape [17,18,19]. This allows the obtaining of sutureless tubular grafts with the desired physical properties and ultrastructure suitable for cell migration and graft vitalization [20,21,22,23,24]. Such scaffolds are expected to resorb and be replaced by a three-layered autologous vessel within one year. Some studies showed that polymeric scaffolds that remain un-resorbed in the organism for longer are subjected to mineralization and fibrosis [25].

Calcification is one of the most frequent complications of vascular grafting. This problem is not specific to artificial scaffolds but is also relevant to xenogeneic and allogeneic materials [26,27,28,29,30,31]. Scientists have been facing implant mineralization in different animal models, with devices made of different materials [31,32,33]. This is also true for polymeric scaffolds, including those made of PCL. The mechanisms of graft mineralization are not fully understood yet, but the importance of their understanding is obviously valuable for the development of organ engineering.

Understanding the normal tissue response and the pathway of post-implantation complications is crucial for discovering the optimal approach in tissue engineering.

This study is a continuation of our previous research where we analyzed the same type of scaffolds and their transformation over shorter periods of time [34]. Hence, we aimed to describe the outcomes of electrospun PCL grafts in a rat abdominal aorta replacement model after a 180-day follow-up and reveal the advantages, disadvantages, and distant effects of these biodegradable constructions.

## 2. Materials and Methods

### 2.1. Polymer Composition

For working solution preparation, 1 g of 3-mm spherical 80 kDa ε-polycaprolactone pellets (cat. № 440744, Sigma-Aldrich Co., St. Louis, MO, USA) were dissolved in 9 g of pure chloroform (Vekton, Saint Petersburg, Russia) using a laboratory shaker (Biosan, Riga, Latvia) at room temperature at 10 rpm for 1 h. Each portion of the solution was prepared ex tempore on the date of electrospinning.

### 2.2. Vascular Scaffold Fabrication

Cylindrical matrices were electrospun at the environmental temperature of 25 °C, and a humidity of 21–22% in a NANON 01-B electrospinning setup (MECC Inc., Fukudo Ogori-shi, Japan) using a clip spinneret and a 2-mm stainless steel rotary collector. The electrospinning protocol was developed by us earlier [34,35]. The setup parameters were: a needle diameter of 27G, a spinneret speed of 150 rpm, a tip-to-collector distance of 150 mm, a collector rotation speed of 300 rpm, a feed rate of 0.5 mL/h, a solution volume of 0.125 mL, an applied voltage of 16 kV, and a nozzle cleaning interval of 59 s.

### 2.3. Scanning Electron Microscopy (SEM)

All the samples were sputtered with a conductive carbon layer of 25–30 nm on a GVC-3000 Thermal Evaporation Carbon Plating Instrument (KYKY TECHNOLOGY Co., Ltd., Beijing, China).

SEM and EDS analysis, and elemental mapping of scaffold and dried explanted grafts were carried out using a WIN SEM A6000LV scanning electron microscope (KYKY TECHNOLOGY Co., Ltd., Beijing, China) equipped with an EDX system AzTec One (Oxford Instruments, Oxford, UK). Specimens were fixed on a specimen stub with conductive tape. Sample observation was conducted using a secondary electron (SE) detector at an electron high tension of 20 keV and electron beam setting of 120 µA. Ten observation fields of every sample were selected and examined at 100×, 250×, 450×, 700×, 800×, and 1000× magnification, then ten 700× fields of view were selected at each sample for the fiber size measurements. The sizes of observed objects were measured using a KYKY SEM Microsoft operating program v. 1.8.1.2.

### 2.4. Graft Sterilization

**Figure 1:**
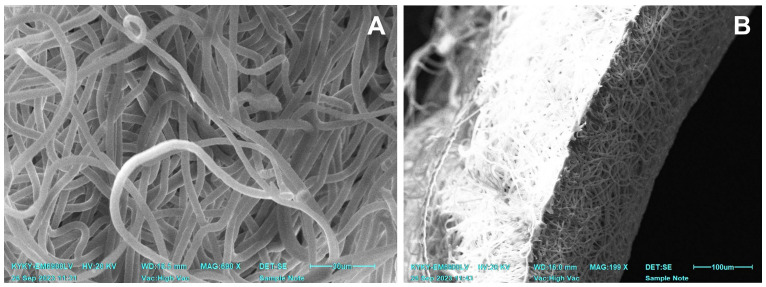
SEM images of a non-implanted one-layered electrospun PCL graft after sterilization. (**A**) A view of PCL nanofibers. (**B**) An image of a scaffold cross-section.

All the tubular scaffolds were sterilized with ethylenoxide (750 mg/L) in a Steri-Vac 5XL sterilizer (3M, St. Paul, MN, USA) at a chamber temperature of 37 °C, an air humidity of 70%, a sterilization cycle time of 3 h, and an aeration period of 8 h. As sterilization is performed at 37 °C, it does not affect the structure of the scaffolds (Figure 1).

### 2.5. In Vivo Experiments

All experimental procedures were carried out in accordance with the EU Directive 2010/63/EU for animal experiments and were approved by the Ethics Committee of E. Meshalkin National Medical Research Center (protocol № 3, 15 June 2021).

Six 450–500 g healthy Wistar male rats were chosen for the experiment. On the day before surgery, the animals were placed in individual cages with free access to drinking water and deprived of food.

#### 2.5.1. Premedication

Premedication included sedation with dexmedetomidine (0.1 mg/kg) (Dexdomitor, Orion Inc., Espoo, Finland) via intramuscular injection in the hind limb. Carprofen (5 mg/kg) (Ricarfa, KRKA d.d., Novo Mesto, Slovenia) was injected for analgesia. Atropine sulfate (0.05 mg/kg) (Dalhimpharm, Khabarovsk, Russia) was administered subcutaneously. The lateral tail vein was catheterized with a 24G catheter (KDM, Berlin, Germany) for intravenous access.

#### 2.5.2. Preoperational Procedures

After the premedication, the fur was shaved off in the abdominal area with a trimmer (Moser, Unterkirnach, Germany), then the animal was fixed on the operation table on its back. Sevoflurane (3%) (Medisorb, Perm, Russia) was administered via the nasal mask of a gas-flow anesthesia system (Ugo Basile, Gemonio, Italy) with an airflow of 0.5 L/min. The working field was swabbed with a 10% povidone-iodine solution (Matyas kiraly ut 65, Kormend, Hungary) and 95% alcohol (Kemerovo Pharmaceutical Factory, Kemerovo, Russia).

#### 2.5.3. Surgery

**Figure 2:**
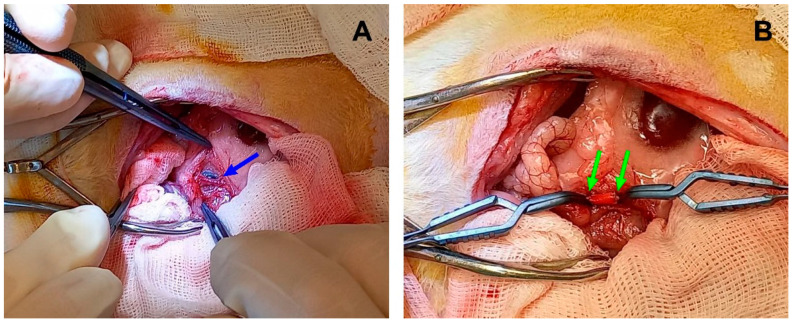
Implantation in rat abdominal aorta. (**A**) Native aorta before sectioning, the blue arrow indicates the vessel. (**B**) An implanted electrospun graft between two vascular clips. The green arrows indicate the margins of a PCL scaffold.

The operation was performed using midline laparotomy and 0.5% lidocaine (1 mL) (Dalhimpharm, Khabarovsk, Russia) was poured intraperitoneally. If muscular contractions of the abdomen occurred, the dosage of lidocaine was increased as necessary but not exceeding a total of 3 mL. The intestine was moved, wrapped in a sterile wet gauze, and washed with a 9% NaCl solution (Solopharm, St. Petersburg, Russia). The abdominal aorta was dissected from the renal arteries to the bifurcation. Fraxiparine (25 IU) (Aspen Notre-Damme de Bondeville, Notre-Damme de Bondeville, France) was injected subcutaneously and then a two-minute pause was held. Then a full aorta occlusion was performed where the upper clip was placed past the renal arteries and the lower clip was placed above the bifurcation. A 5-mm section of the abdominal aorta was removed and an end-to-end anastomoses between the sections and a PCL graft (5 mm length × 2 mm inner diameter) were performed with Optilene 8/0 threads (Braun, Rubi, Spain). (Figure 2B). A total of 1 mg/mL Cefazoline was administered in solution, intraperitoneally (1 mg/kg) (Biosintez, Penza, Russia). The operation wound was closed with a monofilament Optilene 5/0 thread (Bbraun, Rubi, Spain), and the cutaneous suture was swabbed with a povidone-iodine solution.

#### 2.5.4. Postoperative Period

Throughout the entire postoperative period (180 days), all the animals were housed in individual cages with free access to drinking water and food. Postoperative treatment included fraxiparine (15 IU) subcutaneously for three days after the operation, and carprofen (5 mg/kg) subcutaneously for two days after the operation. The surgical outcome was evaluated daily, with specific attention to the overall daily activity, movement in hind limbs, defecation, and urination. Euthanasia was preceded by angiography and ultrasound diagnostics. Before the procedures, all the animals were sedated with dexmetamidine and catheterized with a 24G catheter as described above.

### 2.6. Angiography

CT scans were obtained using a small animal radiation therapy platform (SmART+, Precision X-ray Inc., North Branford, CT, USA). An X-ray tube with dual focal spot sizes (60–80 kVp, small focus with 1-mm Al filtration) was used for imaging. The imaging system is provided with a flat panel amorphous 20 cm × 20 cm (1024 × 1024 pixel) silicon detector set in the opposite position to the X-ray source. For each animal, two series of CBCT scans, with and without the contrast, were performed. To enhance the vessel visualization, the Ultravist 370 contrast medium (732 mg/kg) (Polysan, St. Petersburg, Russia) was injected continuously via the tail vein prior to (15 s) and during (10 s) CT imaging that continued for 50 s.

### 2.7. Ultrasound Diagnostics

Before the procedure, the abdominal area was shaved with a veterinary trimmer and then the Mediagel ultrasound gel (Gelteck, Moscow, Russia) was applied. The ultrasound procedures and Doppler measurements were carried out using a Phillips CX50 portable vascular ultrasound machine (Phillips, Bothell, DC, USA) with a linear transducer. Peak systolic (PSV), end-diastolic (EDV), and time average (TAV) blood flow velocities were valued in three topographic points such as the abdominal aorta above the proximal anastomosis; the midline of the graft and abdominal aorta between the distal anastomosis and bifurcation.

### 2.8. Euthanasia and Autopsy

The animals were euthanized with an overdose of sevoflurane. The abdominal aorta fragments containing the graft were excised via abdominal access and washed in 0.9% NaCl.

### 2.9. Histology

All the samples were fixed in a 10% buffered formaldehyde solution (Biovitrum, St. Petersburg, Russia) for 72 h, then paraffinized in an automatic histological processor (MT Point, St. Petersburg, Russia) with Histomix X-tra paraffin (Biovitrum, St. Petersburg, Russia) and embedded in paraffin tissue blocks in an embedding station (MT Point, Russia, St. Petersburg). Sections of 6-µm thickness were made using a rotary microtome with a heated water bath HM 340 E (Microm, Waldorf, Germany). Obtained sections were stained with hematoxylin and eosin (Biovitrum, St. Petersburg, Russia) following a standard protocol. For specific Von Kossa staining (Biovitrum, St. Petersburg, Russia), staining kits were used according to the manufacturer’s instructions. Light microscopy was carried out at ×40, ×100 and ×400 magnifications using an Olympus CX31 (Olympus, Tokyo, Japan) laboratory microscope with LCmicro 2.2 image analysis software (Olympus Soft Imaging Solutions GmbH, Münster, Germany).

### 2.10. Immunohistochemistry

IHC staining was performed with the use of S 100 (4C4.9) antibodies (Cell Marque, Rocklin, CA, USA), and a referring detection kit (Cell Marque, Rocklin, CA, USA) according to the manufacturer’s instruction.

## 3. Results

### 3.1. Non-Implanted Scaffolds

Non-implanted electrospun scaffolds had an average fiber diameter of 4.39 ± 0.47 µm and a graft wall thickness of 0.12 ± 0.01 mm. In SEM images, all the scaffolds had the same pattern of interlacing loops of nanofibers (Figure 1) throughout the whole sample surface. After the sterilization, the tubular matrices saved their shape and structure.

The scaffolds’ sustainability, patency, and impermeability were confirmed using the test with flowing rat blood (Figure S2).

### 3.2. Surgical Outcomes

All the implanted scaffolds were sustainable, and no signs of rupture, layering, or leakage were noted during or shortly after surgery.

Within the postoperative period, no death or severe surgery complications were observed. None of the animals showed signs of motion impairment or limitation in hind limbs. Overall motion activity decreased in most animals on the first day after the operation and was restored by the next day. The morning after the operation, two rats showed behavioral signs of pain, which was relieved by carprofen injections.

**Figure 3:**
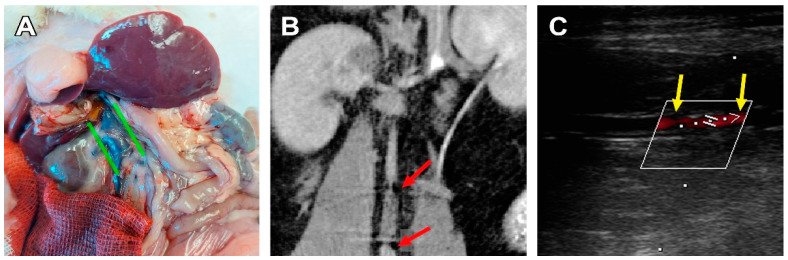
A case of graft stenosis 180 days after implantation. (**A**) Post-mortem examination, green lines indicate the caudal vena cava dilatation. (**B**) CBCT, coronal section, the red arrows indicate areas of insufficient contrast flow at the graft/vessel anastomoses. (**C**) An ultrasonic image of a stenotic graft, frontal section, the yellow arrows indicate the margins of the graft, and the red shadow indicates an area of insufficient blood flow.

Three cases of aorta/scaffold mismatch were discovered when the graft diameter (2.0 mm) was larger than the vessel (1.7–1.9 mm). In two cases this led to in vivo graft deformation, such as graft wall invagination or scaffold shrinkage. These animals showed signs of severe graft deformation and calcification, and anastomotic stenosis, but no leakage, aneurysms, suture ruptures, or thromboses were found on ultrasound and CT images (Figure 3). In these rats, graft stenosis also resulted in compensatory v. cava dilatation. The third animal with a graft mismatch did not show any signs of prosthesis deformation or stenosis as the aortic wall dilated to fit the graft’s diameter. The graft was patent and circular in the transversal section.

**Figure 4:**
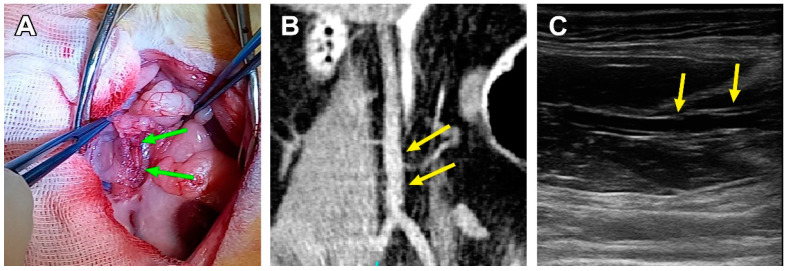
A patent PCL graft sample 180 days after implantation. (**A**) *Post-mortem* examination, green arrows indicate the margins of a PCL graft. (**B**) CBCT, coronal section, the yellow arrows indicate the margins of the graft. (**C**) An ultrasonic image of a patent scaffold, frontal section, the yellow arrows indicate the margins of the graft.

The three other animals showed structural integrity, luminal patency, and the non-deformed tubular shape of the scaffolds, which was confirmed by ultrasonic and macroscopic examination (Figure 4).

### 3.3. PCL Scaffold Transformation

**Figure 5:**
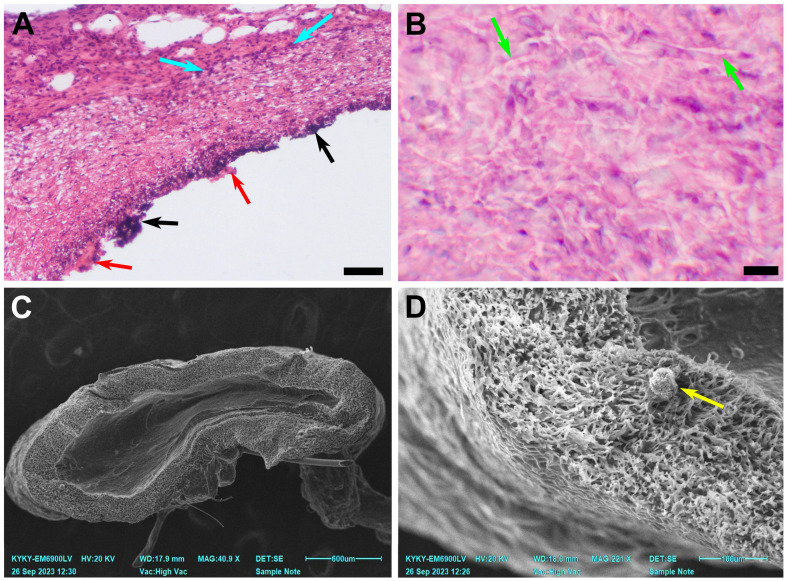
A case of graft stenosis 180 days after implantation. (**A**) A microscopic image of a prosthesis, H&E staining, ×100, bar 50 µm. (**B**) A microscopic image of a prosthesis, H&E staining, ×400, bar 10 µm. (**C**) An overview SEM image of a prosthesis cross-section, bar 600 µm. (**D**) An SEM image of the explant. The green arrows indicate preserved nanofibers, the blue arrows indicate inflammatory cell infiltration, the black arrows indicate mineral clusters, and the red arrows indicate ruptured neointima. A yellow arrow indicates a sputtering artefact.

By day 180, three main types of cell response were observed. The first one coincided with the original graft/vessel diameter mismatch and in vivo graft deformation. Explants from these 2 animals demonstrated a persisting inflammatory response in combination with a preserved fibrillary structure of the scaffold and calcification. Mineral deposits were found either subintimally or between the deep layers of neointima. Regardless, a confluent layer of neointima developed on the luminal surface of the prostheses. In one of the two cases, a severe subintimal mineralization process was discovered (Figure 5). For these explants, the mean PCL fiber diameter was 2.77 ± 0.59 µm.

**Figure 6:**
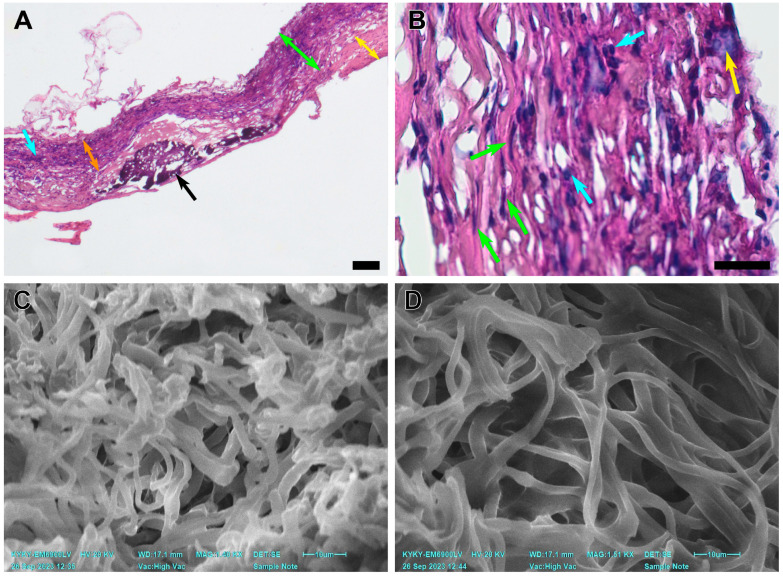
An explanted PCL graft 180 days after implantation. (**A**) A microscopic image of a scaffold, H&E staining, magnification ×40, bar 50 µm. The green double arrows indicate the graft wall, the blue arrows indicate cell infiltration, the black arrows indicate subintimal mineral clusters, the yellow double arrows indicate neointimal layers, and the orange double arrows indicate a revitalized area of the graft. (**B**) A microscopic image of a prosthesis, H&E staining, ×400, bar 10 µm. The green arrows indicate migrating fibroblasts, the blue arrows indicate inflammatory cell infiltration, and the yellow arrow indicates a giant cell. (**C**) An SEM image of a prosthesis cross-section close to the luminal surface. (**D**) An SEM image of a prosthesis cross-section close to the adventitial surface.

The second type of response was found in 3 cases in the group. It implied a permeable, in-grown graft, without significant deformation. These vessels are richly infiltrated with fibroblasts, migrating from the adventitial surface of the scaffold to the lumen which is covered with thick hyperplastic neointima layers. A small to medium inflammatory cell infiltration was observed, and giant cells were revealed occasionally (Figure 6).

Notably, the PCL fibers on the luminal side (from 2.39 to 3.40 µm) of these grafts were thicker than those on the adventitial side (from 1.62 to 1.90µm). The total average fiber thickness in these samples was 2.29 ± 0.42 µm. The adventitial surface of these scaffolds was more integrated into adjoining tissues, and its pores were filled with cellular components and interstitial medium. The pores on the luminal surface of these grafts were filled partially and cell content was less featured in this part (Figure 6).

**Figure 7:**
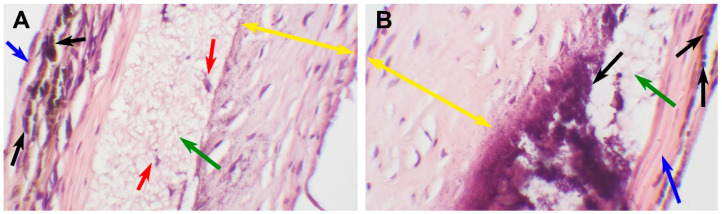
An encapsulated explanted PCL graft 180 days after implantation. (**A**) A microscopic image of a scaffold, H&E staining, ×100, bar 50 µm. The green arrow indicates preserved nanofibers, the red arrows indicate single-cell infiltration, the blue arrow indicates an external fibrotic capsule, the black arrows indicate mineral clusters, and the yellow double arrows indicate neointima. (**B**) A microscopic image of a prosthesis, H&E staining, ×400, bar 10 µm. The blue arrow indicates an external fibrotic capsule, the black arrows indicate mineral clusters, and the yellow double arrows indicates neointima, the green arrow indicates preserved nanofibers.

The third type of reaction was represented by a single case of complete graft encapsulation (Figure 7).

This type of response is characterized by a fibrillary matrix structure with extremely poor cell infiltration. The partially degraded scaffold is surrounded by fibrotic tissue; a thick layer of hyperplastic neointima on the luminal surface and a dense fibrotic capsule on the adventitial surface. The implant was patent and functionally sustainable throughout the observation period; however, it showed the least signs of biodegradation. The fibers in this scaffold insignificantly differed from the original matrix.

**Figure 8:**
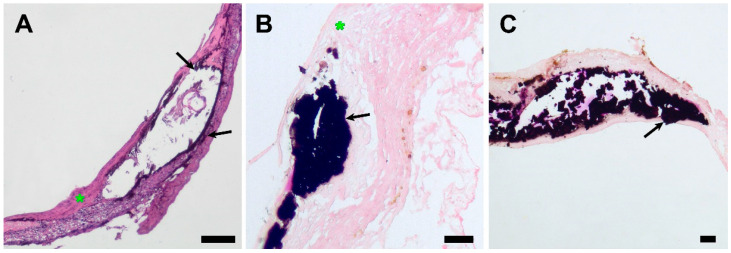
Light microscopy images of explanted PCL grafts 180 days after implantation. (**A**) H&E staining, ×40, bar 50 µm. (**B**) Von Kossa staining, magnification ×100, bar 10 µm. (**C**) Von Kossa staining, magnification ×40, bar 50 µm. The black arrows indicate subintimal mineral clusters, and the green asterisk indicates neointimal layers.

All the examined grafts demonstrated neointimal hyperplasia and calcification loci (Figure 8).

**Figure 9:**
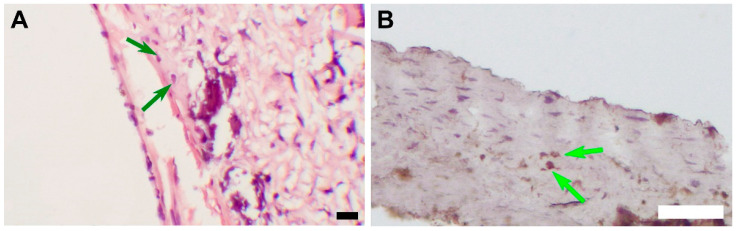
(**A**) An image of an H&E-stained section, magnification ×400, bar 10 µm. The green arrows indicate cells with morphological signs of chondroid metaplasia. (**B**) An image of an IHC-stained section, magnification ×100 bar 50 µm. The green arrows indicate S 100 positive cells with morphological signs of chondroid metaplasia.

Mineralization sites were located subintimally, except for an encapsulated sample, where mineral clusters were found both on the luminal surface of the scaffold and in the external capsule (Figure 7). A widespread mineralization that caused partial splitting of the neointima during tissue processing was found in one of the grafts with luminal stenosis. (Figure 5) The profound layers of neointima contained cells that showed morphological signs of chondroid metaplasia, positive for S 100 proteins IHC staining (Figure 9).

**Figure 10:**
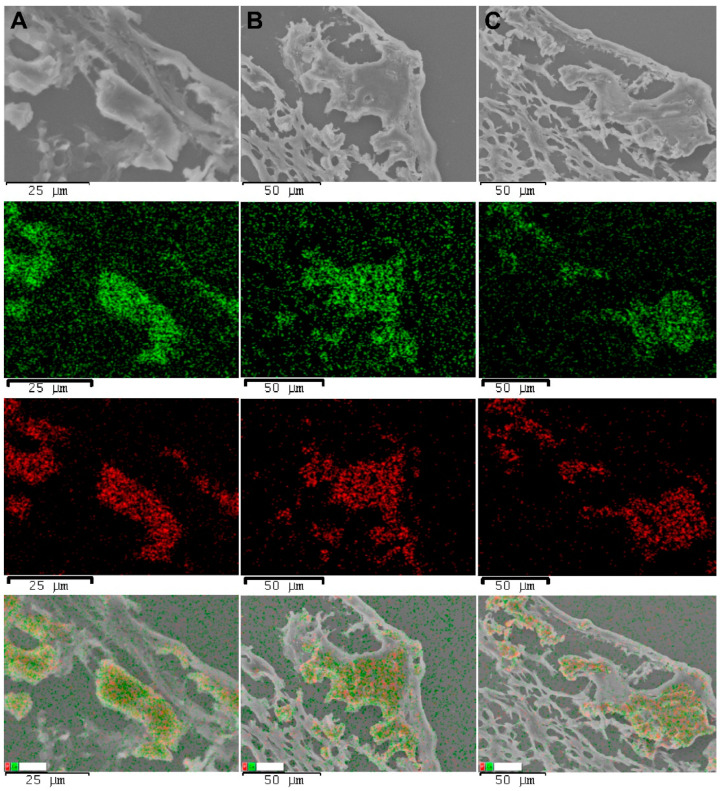
SEM images of explanted PCL grafts, element maps, and an overlay map, 180 days after implantation. Row (**A**), a stenotic graft with inflammation. Row (**B**), a patent graft with a remodeling process. Row (**C**), an encapsulated graft. The green points indicate calcium signals, and the red points indicate phosphorus signals.

SEM imaging and element mapping confirmed the presence of subintimal mineral deposits in all the samples (Figure 10).

According to the EDS analysis, these formations mostly consisted of calcium and phosphorus.

## 4. Discussion

Our previous study [34] described the surgical outcome of similar vascular grafts in shorter time periods. Regarding that experience, we have met several practical aspects that could be improved. Solving these challenges, we increased the graft wall thickness 2 times, from 54.10 ± 7.37 µm to the current size, which facilitated the surgical procedures and improved the fabrication process. Thicker scaffolds are less likely to crumple and deform during implantation and are easier to remove from the collector. According to the CBCT and ultrasonic data, it did not affect their in vivo performance. The non-woven matrix architecture and the nanofiber structure remained unchanged.

Positive overall surgical outcomes of the implanted grafts confirmed PCL as a sustainable scaffold material with acceptable durability and mechanical properties corresponding to a sufficient minimum of physical properties. It is also an easily processed and affordable polymer. The obtained grafts were comfortable for storage and surgical use. At the same time, its long-term in vivo performance is associated with several crucial disadvantages.

There is plenty of research considering synthetic materials for vascular grafting [1,2,27,28,29,30,31,32]. However, opinions on the importance of complete resemblance of native vessel biomechanics before the graft vitalization were divided. Since a biodegradable scaffold is meant to be replaced by the recipient’s autologous cells within a year, rigorous demands for identical mechanical properties might be secondary to the remodeling process. On the contrary, some scientists focused on graft remodeling as the core subject of the scientific search in this field [22,25]. From this point of view, PCL matrices have significant limitations before some of the well-known polymers offered for use in tissue engineering [36,37]. For example, it has lower tensile strength compared to PGA and PLA [37] and a slow biodegradation rate [25,38].

H. Sun and coauthors [38] observed the degradation of PCL capsules for three years and declared that the implanted pieces remained intact for as long as two years. Notably, in this research, the devices were solid pieces made of the polymer with a relatively low molecular mass of 66 kDa, while we used a stronger polymer (80 kDa) in search of the desired physical properties and fiber structure, and easier processing due to higher polymer chain entanglement. Assumingly, its degradation would take even longer. However, the electrospun scaffold’s nanostructure is a significant advantage, as the graft is permeable for cells, as opposed to the solid structures. The contact and, therefore, the reactive surface between the polymer and the internal environment greatly increased. This property of porous grafts is aimed to balance the high molecular mass of the polymer. These differences also impose limitations in comparison with our experience.

In our previous research [34], we found that cell migration in the scaffolds gradually increased up to 60 days of observation, then (90 days) it was followed by a decrease in cell ingrowth. Judging by the cell types, this pattern might be associated with an inflammatory response to the implanted device. Evaluating the 180-day results, we note a featured increase in graft cellularity with a predominance of fibroblasts. In three rats that did not show a persisting inflammatory cell infiltration, stenosis, or graft encapsulation, the adventitial side of the implant was revitalized. As mentioned above, fibers in this part of the graft are thinner than on the less cellularized luminal surface (Figure 6). Interestingly, in the animals showing featured inflammatory cell infiltration, the grafts had a uniform fiber structure and thickness throughout the scaffold. In two cases with graft stenosis, featured lymphocyte infiltration was found, but very few giant cells appeared in the observation fields. The patent grafts with signs of the scaffold remodeling show a different histological picture. Multinuclear giant cells were well represented (Figure 6), which indicated the process of active scaffold degradation. The formation of a fibrotic capsule isolated the scaffold preventing cell ingrowth and, as a result, only singular cells morphologically similar to fibroblasts and macrophages appeared in the observation field. Since this was the only case of graft encapsulation, we associated it with the individual features of an animal and suggested that this histological picture was a result of local inflammation.

Many authors studying PCL grafts pointed out the high risk of neointimal hyperplasia development [16,39,40,41]. According to our results, gained neointima does not affect the grafts’ hemodynamics in the follow-up period, but there are prerequisites for the growth to continue, which may result in graft stenosis, thrombotic complications, and graft failure. Some researchers [16,40,41] found correlations between neointimal hyperplasia and scaffold mineralization. In particular, there are reports about mineral deposits, mainly located subintimally. One of the mechanisms of subintimal graft calcification, suggested in the literature, inquired about the presence of cells with signs of chondroid metaplasia [16,41]. De Valence et al. [16] attributed this transformation to the differentiation of smooth muscle cells on the luminal surface into chondrocytes. To investigate this approach, an S 100 IHC staining was performed [42]. S 100 positive cells with a specific morphology were found in all the samples, except for ones with inflammatory infiltration. In the case of an encapsulated graft, mineral clusters were identified by Von Kossa staining both in the adventitial and under the luminal layers of fibrosis, but S 100 positive cells were found only on the luminal surface of the graft. This finding may be explained by the influence of bioactive substances, highly available in the blood flow. For example, J.-S. Shao and colleagues point out the specific role of osteopontin (OPN) in vascular calcification [43], while Yang et al. [40] highlighted the specific role of exosomes in these processes. Some groups do report the following ossification of the PCL prostheses, which we did not note. Resuming our own findings, we can state that the unrelated foci of both chondroid metaplasia and mineralization were observed in the same samples. Besides, mineral clusters were found in fibrotic tissue that did not contain metaplastic cells, which questions this hypothesis. We also note that the small size of the experimental group puts limitations on this conclusion.

These findings provoke the question of the importance of prompt biodegradation of the scaffold corresponding to its cellularization and revitalization. There is no evidence that the PCL degradation product, 6-hydroxy hexanoic acid [44], influences the vessel patency or the adjoining tissue reactivity, but there are many reports about its long degeneration and unsustainability in long-term performance [16,38,41].

The problem of mineralization does not refer to artificial grafts solely, but it also affects other types of vascular prostheses. It was described for various vessel and valve materials including xenogeneic tissues, decellularized biomaterials, and synthetic substances. Metaplastic processes involving chondroid transformation and ossification were noted in many animal models [16,45] and humans [46]. The variability of the conditions leading to graft calcification suggests that the difference in response does not rely just on the substance of implants but can be an imminent consequence of a vessel impairment in combination with a foreign body reaction.

## 5. Conclusions

In this study, we have described the long-term outcomes of a PCL graft in the rat abdominal aorta 180 days after implantation. We have identified three main types of observed histological reactions, which are: graft encapsulation, chronic inflammation, and scaffold degradation with remodeling and vitalization. Also, all the implants were subjected to intimal hyperplasia and subintimal mineralization. The gained results vary from data reported by some other groups. In particular, no graft ossification was found at this point, but the presence of S 100 positive cells with signs of chondroid metaplasia was confirmed in profound layers of neointima. In terms of surgical outcomes, grafts, which were not subjected to inflammation, were stable and patent for the whole follow-up period. The grafts with persisting inflammation were stenotic.

Our results suggest the significance and specificity of cell differentiation in remodeling artificial vascular prostheses. The mechanisms of these processes are yet to be studied, but the importance of their disclosure and understanding of the pathways is evident.

## Data Availability

The data presented in this study are available within the article.

## Supplementary Materials

The following supporting information can be downloaded at: https://www.mdpi.com/article/10.3390/polym15214304/s1. Figure S1: A scheme of the experiment, Figure S2: A fabricated vessel, tested for blood permeability, Figure S3: A view of a graft wall and lumen, Figure S4: A paraffin section of an explant, Russel-movat pentachrome staining, Figure S5: Paraffin sections of explants, Russel-movat pentachrome staining.
