# Supplementary material for: Remote Outcomes with Poly-ε-Caprolactone Aortic Grafts in Rats
Source: Polymers (Basel). 2023 Nov 2;15(21):4304. doi: 10.3390/polym15214304 (PMC10649699; doi:10.3390/polym15214304)
Supplement: Supplementary file 1 [file polymers-15-04304-s001.zip › polymers-2667818-supplementary.pdf]

Remote outcomes with poly-e-caprolactone aortic grafts in rats.

Supplementary files.

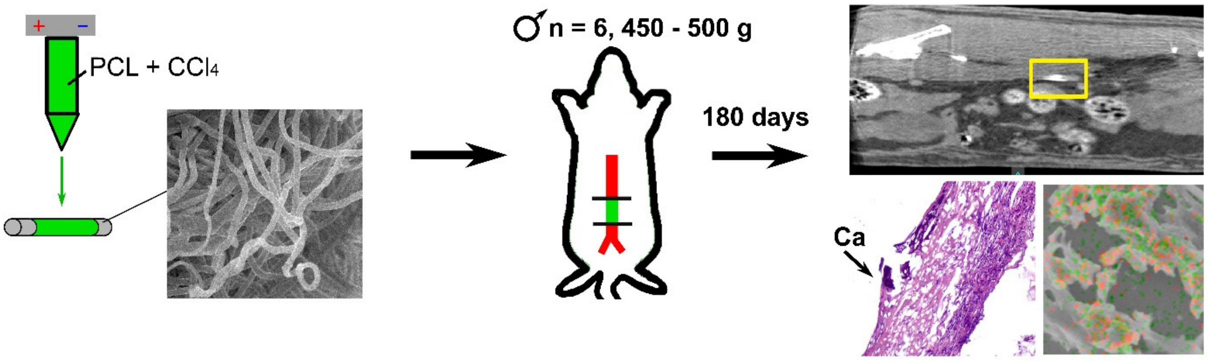

Supplementary Figure S1. A scheme of the experiment: electrospinning of the polymeric solution (10% wt PCL in pure chlorophorm) with a view of obtained fibers, *in vivo* implantation in rats (180 days follow-up), functional diagnostics and microscopic analysis.

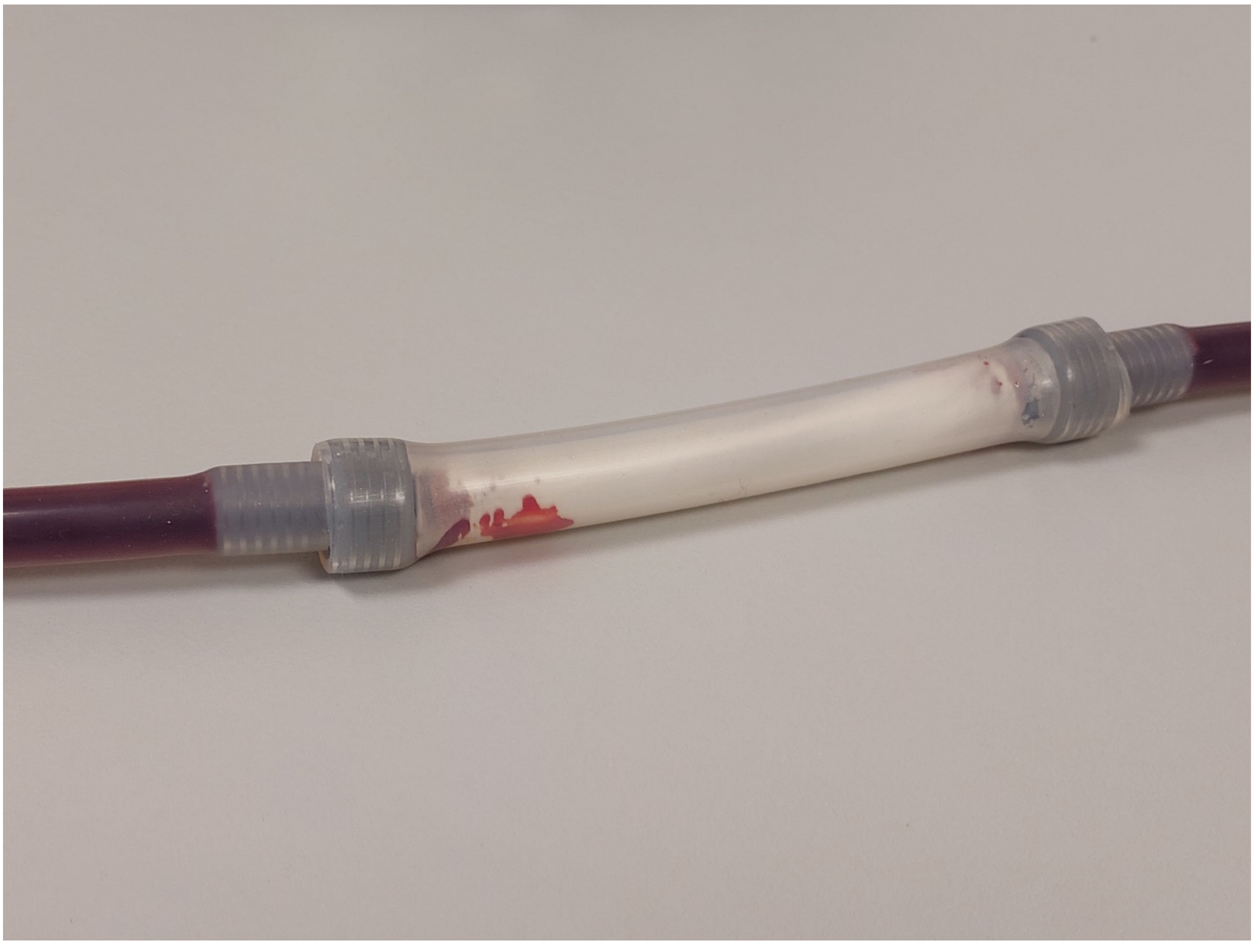

Supplementary Figure S2. A fabricated vessel, tested for blood permeability. The leackage was caused by fixators of the device.

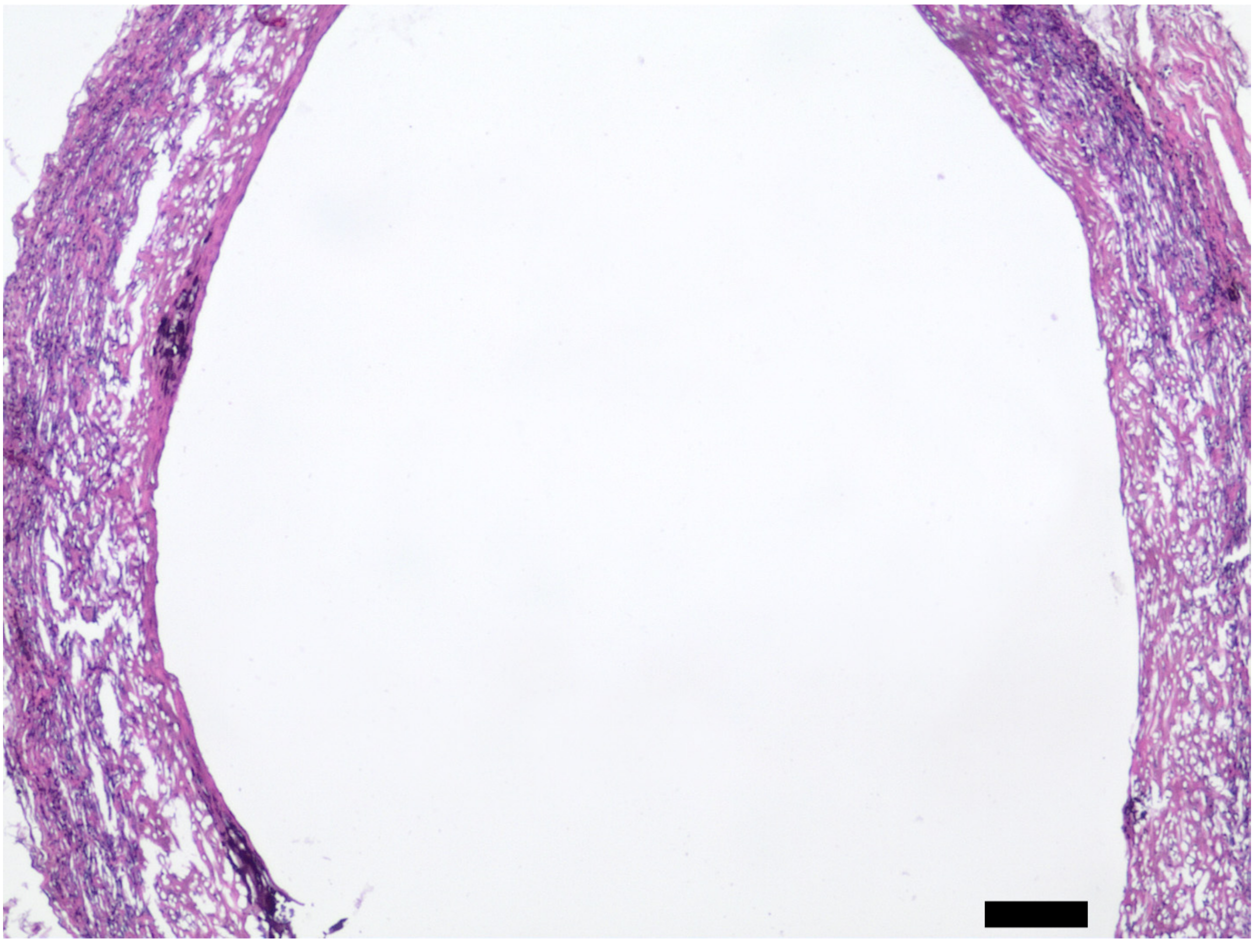

Supplementary Figure S3. A view of a graft wall and lumen, 180 days after implantation. H&E stainig, ×40, bar 50 μm.

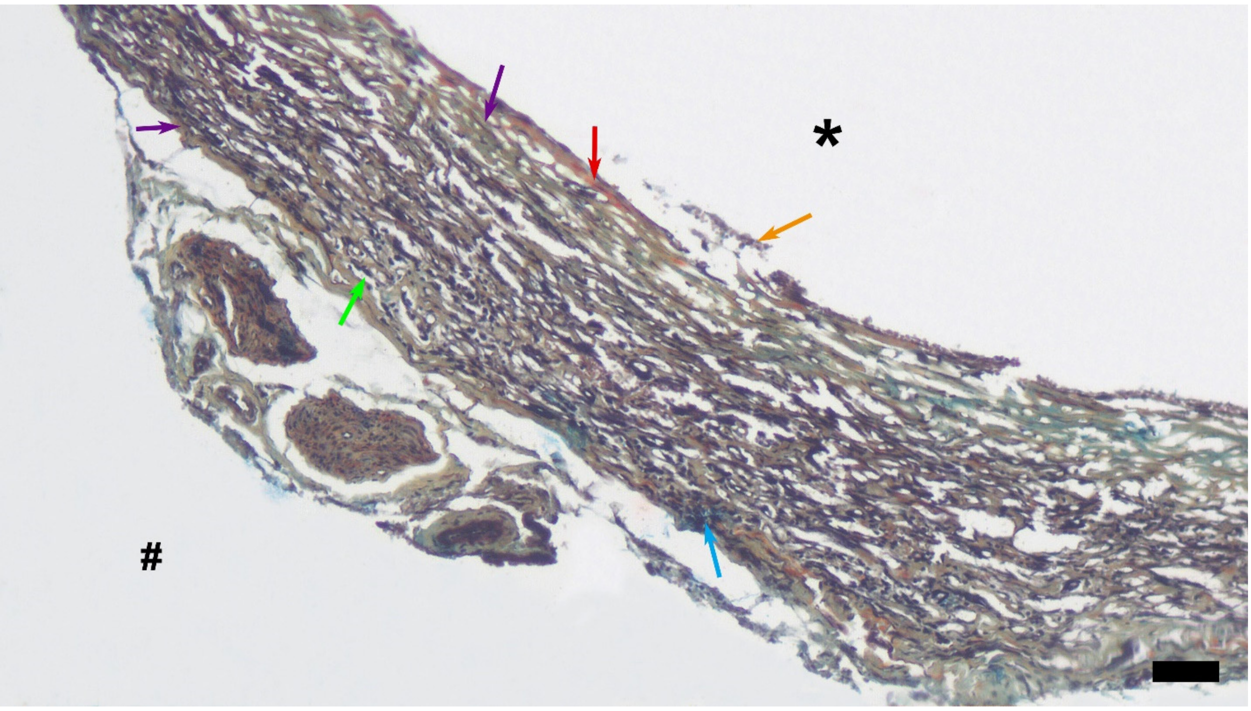

Supplementary Figure S4. A paraffin section of an explant, Russel-movat pentachrome staining. Magnification ×100, bar 50 μm. The asterisk indicates luminal surface of a graft, the number sign indicates the adventital surface, the red arrow indicates smooth muscle component (stained red), the purple arrows indicate

collagen fibers (stained yellow), the blue arrow indicates cellular nuclei (dark purple), the green arrow indicates gathering fibroblastic cells (nuclei stained purple), the orange arrow indicates blood cells foud in the graft lumen.

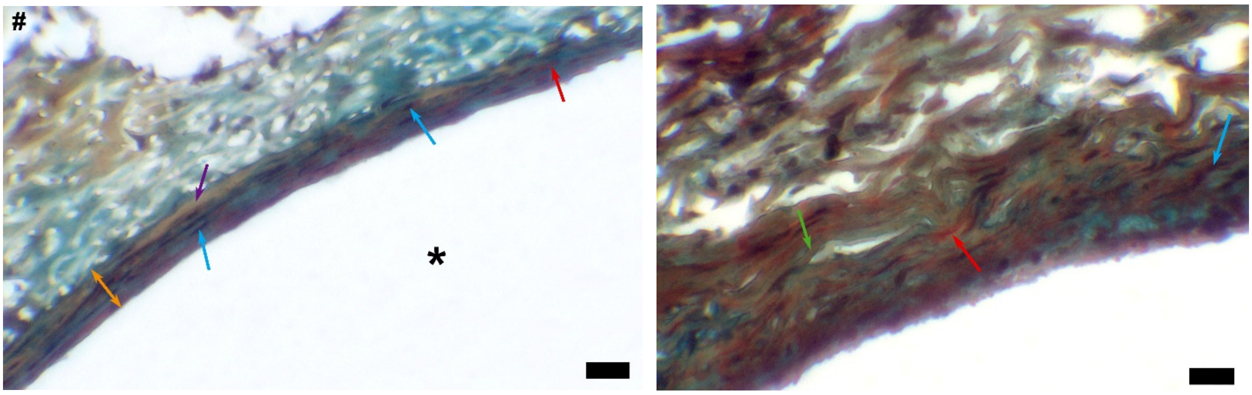

Supplementary Figure S5. Paraffin sections of explants, Russel-movat pentachrome staining. Magnification ×400, bar 10 μm. The asterisk indicates luminal surface of a graft, the number sign indicates the adventital surface, the red arrow indicates smooth muscle component (stained red), the purple arrow indicates collagen fibers (stained yellow), the blue arrows indicate cellular nuclei (dark purple), the green arrow indicates collagen fibers (stained yellow), the orange arrow indicates neointima.
